# Sustaining Electron Transfer Pathways Extends Biohybrid Photoelectrode Stability to Years

**DOI:** 10.1002/anie.202201148

**Published:** 2022-04-19

**Authors:** Vincent M. Friebe, Agata J. Barszcz, Michael R. Jones, Raoul N. Frese

**Affiliations:** ^1^ Department of Physics and Astronomy LaserLaB Amsterdam VU University Amsterdam De Boelelaan 1081 Amsterdam 1081 HV The Netherlands; ^2^ School of Biochemistry Biomedical Sciences Building University of Bristol University Walk Bristol BS8 1TD UK; ^3^ Electrobiotechnology Campus Straubing for Biotechnology and Sustainability Technical University of Munich Schulgasse 22 94315 Straubing Germany

**Keywords:** Biosensors, Electrochemistry, Photochemistry, Photosynthesis, Solar Energy Conversion

## Abstract

The exploitation of natural photosynthetic enzymes in semi‐artificial devices constitutes an attractive and potentially sustainable route for the conversion of solar energy into electricity and solar fuels. However, the stability of photosynthetic proteins after incorporation in a biohybrid architecture typically limits the operational lifetime of biophotoelectrodes to a few hours. Here, we demonstrate ways to greatly enhance the stability of a mesoporous electrode coated with the RC‐LH1 photoprotein from *Rhodobacter sphaeroides*. By preserving electron transfer pathways, we extended operation under continuous high‐light to 33 days, and operation after storage to over two years. Coupled with large photocurrents that reached peak values of 4.6 mA cm^−2^, the optimized biophotoelectrode produced a cumulative output of 86 C cm^−2^, the largest reported performance to date. Our results demonstrate that the factor limiting stability is the architecture surrounding the photoprotein, and that biohybrid sensors and photovoltaic devices with operational lifetimes of years are feasible.

## Graphical abstract

The viability of biohybrid technologies is primarily limited by the inherent fragility of the biological component. The operational stability of photoproteins adsorbed to mesoporous ITO electrodes is prolonged to 33 days under intense illumination, and 28 months in storage, demonstrating the feasibility of protein‐based devices that last years.

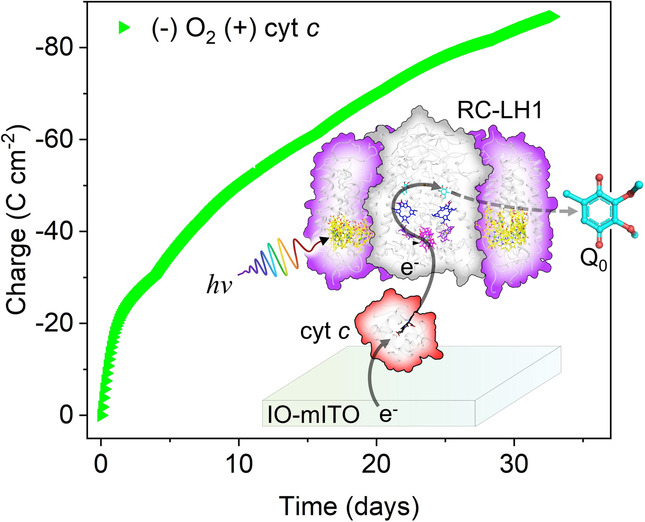

## Introduction

The bulk of the biosphere is powered by solar energy conversion catalyzed by nanoscale photodiodes termed reaction centers (RCs). These pigment‐proteins, found in both eukaryotes and prokaryotes, use either chlorophyll (Chl) or bacteriochlorophyll (BChl) to capture, convert and store solar energy.^[1]^ Although photosynthesis overall is not a particularly energy‐efficient process,^[2]^ the primary events of energy transfer during light capture, and charge separation during energy stabilization, take place with very high quantum efficiencies (events per photon absorbed).^[3]^ The deduced biomolecular origins of this high efficiency have guided the design of man‐made materials for sustainable solar energy conversion,[^3^, ^4^] and also driven interest in the direct integration of photosynthetic complexes in biohybrid technological devices.[^5^, ^6^, ^7^, ^8^]

The three types of photoprotein most commonly used for the fabrication of biohybrid electrodes are the Chl‐containing Photosystem II (PSII) and Photosystem I (PSI) complexes from oxygenic cyanobacteria and plants, and the BChl‐containing reaction center‐light harvesting 1 (RC‐LH1) complexes from anoxygenic photosynthetic bacteria such as *Rhodobacter* (*Rba*.) *sphaeroides*.^[9]^ Recent advances, including the development of transparent high‐surface‐area electrodes that enable substantial photoprotein loading and light penetration,[^10^, ^11^, ^12^, ^13^, ^14^, ^15^] have resulted in some of the highest output semi‐artificial biophotoelectrodes to date. Examples include PSII immobilized on an inverse‐opal mesoporous indium tin oxide electrode (IO‐mITO), which produced photocurrent densities up to 930 μA cm^−2^,[^10^, ^14^] and PSI wired using cytochrome (cyt) *c* to an IO‐mITO electrode that produced photocurrents of 300 μA cm^−2^.^[15]^ *Rba. sphaeroides* RCs deposited on nanostructured silver have generated photocurrents up to 416 μA cm^−2^ in a conventional biophotoelectrochemical cell,^[11]^ whereas RC‐LH1 proteins coupled to photoactive semiconductor materials in solid‐state configurations have shown stable photocurrents up to 1.3 mA cm^−2^,^[16]^ and RC‐LH1 multilayers suffused with gel electrolytes have shown stable photocurrents up to 850 μA cm^−2^.^[17]^ While these current densities are still an order of magnitude away from those of solar cells made from purely synthetic materials, rapid growth in biophotoelectrode performance is closing this gap.[^6^, ^14^, ^18^]

The principal stumbling block to more widespread development of protein‐based solar energy conversion devices is limitations in the stability of current output under operation and following storage.[^18^, ^19^] Reviews of systems based on PSI^[18]^ and PSII[^14^, ^20^] have documented that, where reported, operational stability does not extend past a few hours. The longest reported continuous operation of an aqueous PSI‐based biophotoelectrode is 16 hours when illuminated 30 % of the time.^[21]^ Reported operational stabilities of PSII‐based bioelectrodes are on the order of minutes, the limitation being photodamage arising from the high‐potential water splitting reaction.^[14]^ The BChl‐containing *Rba. sphaeroides* RC carries out charge separation at much less oxidizing and reducing potentials than the Chl‐containing PSII and PSI complexes, respectively, and RC and RC‐LH1 based biohybrid systems have been reported to operate for up to 65 hours under continuous illumination in air.^[22]^ Despite this advantage, operational stability still falls three to five orders of magnitude short of the desired device stability of at least one year.[^18^, ^23^]

In this work, we sought to prolong solar energy conversion by *Rba. sphaeroides* RC‐LH1 complexes (Figure S1) deposited on an IO‐mITO electrode from days to years. We determined that biophotoelectrode activity is primarily limited by the integrity of the electron transfer pathways between the photoprotein and electrode, and that by stabilizing this interface, we could extend operation up to a full month under intense continuous illumination. We also reveal unprecedented stability during storage for over two years under cold, dark conditions, an attribute essential for enzyme‐based applications such as biosensors. These step‐changes in operational and storage stability will help realize viable photoprotein‐based technologies that demand operational stability on the order of years.

## Results and Discussion

**Figure 1:**
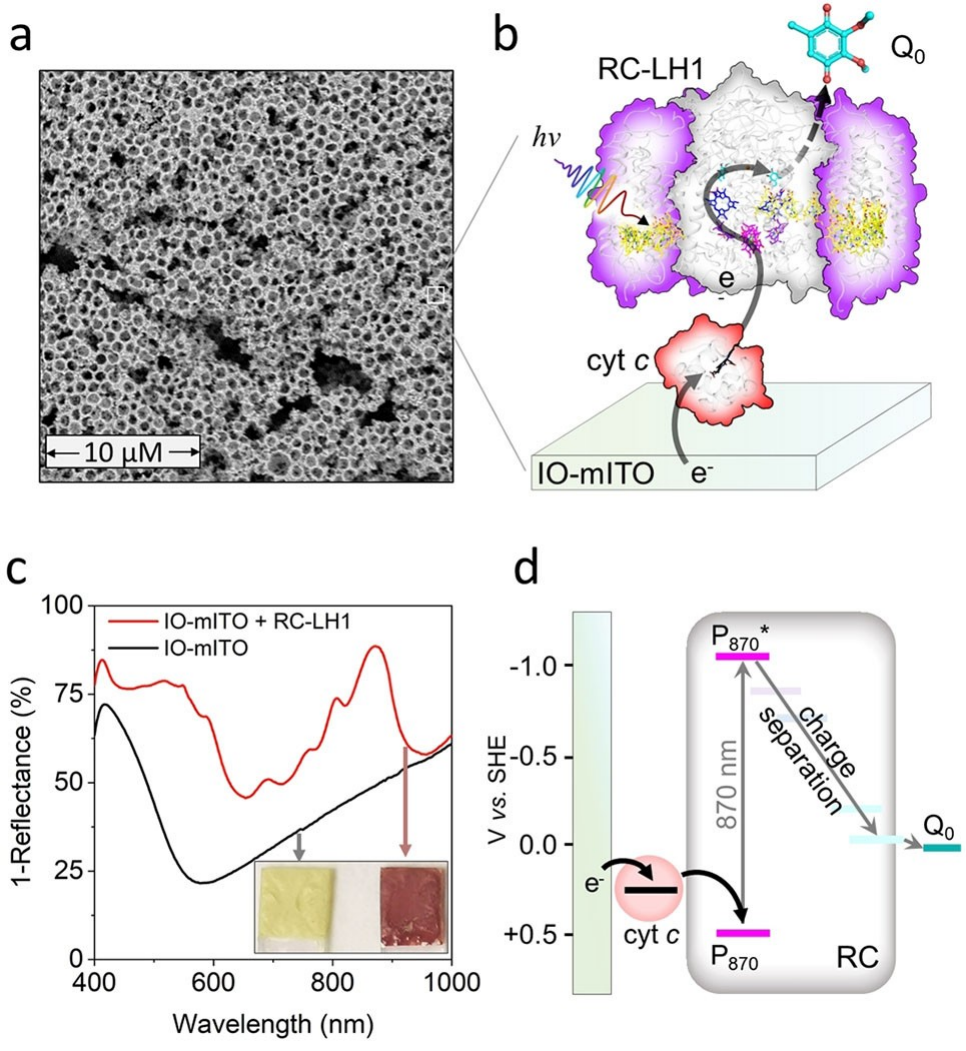
Architecture, mechanism, and loading of the IO‐mITO|cyt *c*|RC‐LH1 electrode. a) SEM image of a section through an IO‐mITO electrode formed using 800 nm diameter polystyrene beads. The white bar represents 10 μm. b) Outline of the electron transfer scheme in the RC‐LH1 biophotocathode. c) 1‐Reflectance spectra of IO‐mITO electrodes with and without loaded RC‐LH1 protein and (inset) images of the electrodes. d) Energy level diagram of the electron transfer pathway.

Using strips of ITO‐glass as a base, 800 nm diameter polystyrene beads and polydispersed ITO nanoparticles were used to create IO‐mITO electrodes as described in the Experimental Section. Scanning electron microscopy (SEM) revealed a structure formed from spherical cavities of ≈800 nm diameter together with larger crevices (Figure 1a). To deposit RC‐LH1 protein (Figure 1b) the electrode surface was cleaned in basic piranha solution to leave behind a hydroxyl‐functionalized,^[24]^ protein‐friendly surface,^[10]^ before multiple rounds of sequential immersion first in a solution of RC‐LH1 complexes followed by a solution of cyt *c*. Successful adsorption of RC‐LH1 complexes onto the IO‐mITO electrode was apparent by eye (inset to Figure 1c). Reflectance spectra of coated electrodes showed distinctive features at 870, 805 and 600 nm attributable to the RC‐LH1 BChls and a broad feature between 430 and 600 nm attributable to the RC‐LH1 carotenoids (Figure 1c). The resulting coated working electrodes were then immersed in a buffer containing 1.5 mM ubiquinone‐0 (UQ_0_) at pH 8.0. The expected energetics of the sequence of electron transfer reactions initiated by light absorption by this system, illustrated schematically in Figure 1b, is shown in Figure 1d. Photoexcitation oxidizes the P_870_ BChl primary electron donor of the RC and reduces its Q_B_ ubiquinone terminal electron acceptor (see Figure S1 for more detail). Cyt *c* wires the oxidized terminal of the RCs to the IO‐mITO electrode while UQ_0_ acts as a mobile carrier of charge to the counter electrode.[^11^, ^25^, ^26^]

**Figure 2:**
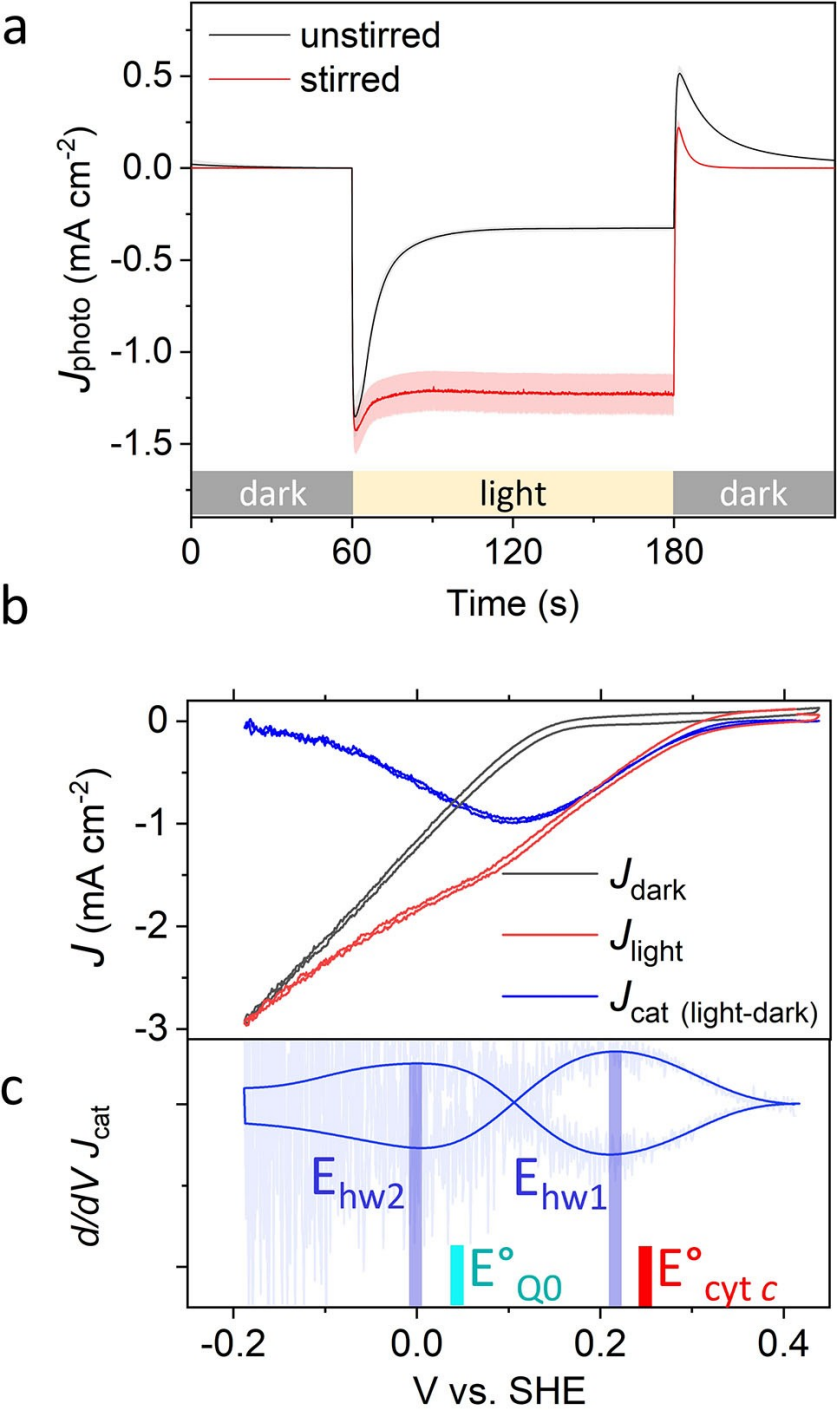
Photochronoamperometry and photocatalytic voltammetry. a) Photocurrent densities from an optimized IO‐mITO|cyt *c*|RC‐LH1 electrode in 20 mM Tris (pH 7.0)/50 mM KCl/5 mM Q_0_ without (black) or with (red) stirring. Shaded regions represent the standard error, *n*=5. b) Photocatalytic voltammograms under stirred conditions in the dark (*J*_dark_—black), light (*J*_light_—red), and the difference (*J*_cat (light‐dark)_—blue). c) Derivative of *J*_cat_ overlaid with a Fourier transform filtered smooth with a cut‐off at 0.045 Hz to remove fluctuations from stirring (dark blue). Half‐wave potentials (*E*_hw_) are indicated by the blue bars. Formal potentials (*E* ^0^) of Q_0_ and cyt *c* are indicated by cyan and red bars, respectively.

The *J*_peak_ produced by illumination of such IO‐mITO|cyt *c*|RC‐LH1 electrodes was used as a metric for optimization of the thickness of the IO‐mITO layer and amount of protein loaded. Experiments with between one and five sequential layers of IO‐mITO showed that three such layers and four to six rounds of protein loading were optimal (Figure S2a, b). This configuration produced an average *J*_peak_ of 4.2±0.2 mA cm^−2^, with a best‐performing electrode producing 4.6 mA cm^−2^. After this initial peak, the photocurrent declined sharply to a stable level (*J*_stable_) of around 0.2 mA cm^−2^ (Figure S2c). Since solar energy conversion requires performance under continuous light conditions, further optimization of *J*_stable_ was pursued. A mean *J*_stable_ of 0.32±0.03 mA cm^−2^ was obtained by increasing the concentration of UQ_0_ to 5 mM and changing the electrolyte from pH 8.0 to pH 7.0 (Figure S3 and Figure 2a). The increase in *J*_stable_ at pH 7.0 vs pH 8.0 likely stems from the shifted pH dependent ubiquinone midpoint potential (+59 mV per decrease in pH unit).^[27]^ Decreasing the pH by one unit effectively lowers the driving force for charge recombination between ubiquinol and the electrode.^[28]^ These buffer conditions were used for the remainder of the study, and the data below are for an optimal electrode formed from three layers of IO‐mITO, which produced a ≈69±23 μm thick working electrode matrix (Figure S2d), and four rounds of protein deposition.

Evidence that the marked decline from *J*_peak_ to *J*_stable_ was caused by mass transport limitations was provided by stirring the buffer, which largely removed this decline (Figure 2a and Table 1). Stirring also largely removed the reverse current spike seen when illumination ceased (Figure 2a; at 180 s), which has been attributed to charge recombination at the electrode surface.^[28]^

**Table 1:** Performance of IO‐mITO|cyt *c*|RC‐LH1 electrode.

	(−) Stirring	(+) Stirring
*J*_peak_	1367±270 μA cm^−2^	1486±286 μA cm^−2^
*J*_stable_	322±29 μA cm^−2^	1230±110 μA cm^−2^
Γ_RC‐LH1_	272±27 pmol cm^−2^	272±27 pmo cm^−2^
Γ_cyt *c* total_	6.23±0.7 nmol cm^−2^	6.23±0.7 nmol cm^−2^
Γ_cyt *c* electroactive_	6.15±0.4 nmol cm^−2^	6.15±0.4 nmol cm^−2^
*k*_cyt *c*_	7.8 s^−1^	–
^[a]^TOF_app_ (*J*_stable_)	12.3±2 e^−^ s^−1^ RC^−1^	47±6 e^−^ s^−1^ RC^−1^
^[a]^IQE_app_ (*J*_stable_)	1.6±0.2 %	6.3±0.8 %

Standard errors are reported, *n*=4. TOF and IQE are calculated from *J*_stable_, as indicated in parentheses. [a] values are considered apparent values since loss processes such as short‐circuiting or inactive RC‐LH1 s are not considered.

While *J*_stable_ under stirring was large, the apparent RC‐LH1 turnover frequency (TOF_app_) reached a value of only 47 e^−^ s^−1^ (Table 1), which is far below the maximally observed TOF of ≈1250 e^−^ s^−1^ in vitro,^[29]^ and ≈500 e^−^ s^−1^ in situ^[30]^ (Table S1). We hypothesize that the relatively low TOF_app_ is partially a consequence of the high protein loading, which dilutes the incident photon flux as it penetrates the electrode, leading to a lower average RC‐LH1 excitation rate due to self‐shading. A mean electrode RC‐LH1 loading (Γ_RC‐LH1_) of 272±25 pmol cm^−2^ was determined by solvent extraction of the BChl *a* pigment of adhered RC‐LH1 complexes (Figure S4a). Using the known extinction coefficient of RC‐LH1 at 874 nm,^[30]^ the loaded protein was calculated to absorb 88 % of incident photons at this wavelength, in close agreement with the measured 1‐Reflectance of 91 % at the same wavelength (Figure 1c).

The loading of RC‐LH1 complexes in the IO‐mITO matrix represents at least a 20‐fold increase relative to an RC‐LH1 coated nanostructured silver electrode described previously,^[11]^ explaining a 10‐fold increase in *J*_peak_ relative to our previous work (Table S1). The distribution of RC‐LH1 on the surface layers of the IO‐mITO electrode was investigated by confocal fluorescence microscopy. An image of a 10×10 μm area (Figure S5a) showed peaks and troughs of RC‐LH1 fluorescence approximately 0.8 μm in size, in agreement with the size of the inverse opal voids (Figure S5b). The loading of cyt *c* (*Γ*_cyt *c*_) was found to be in 20‐fold molar excess relative to the RC‐LH1 complex (Figure S4b). This ratio was guided by previous studies which demonstrated that a large pool of cyt *c* is necessary to efficiently funnel electrons from an electrode surface to adsorbed RCs.^[25]^

**Figure 3:**
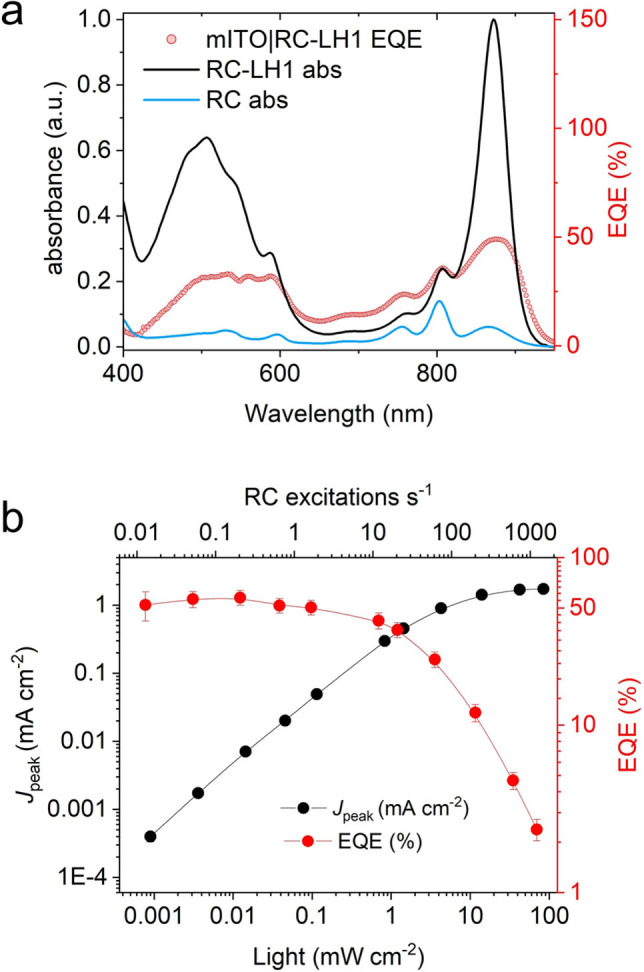
EQE action spectra. a) Percentage external quantum efficiency, determined from photocurrent transients recorded with 2 nm increments of excitation wavelength, compared with the RC‐LH1 absorbance spectrum. The spectrum of a pure RC is shown to distinguish LH1 contributions. b) *J*_peak_ and percentage EQE as functions of illumination intensity and RC turnover frequency (excitations s^−1^).

The electron transfer mechanism depicted in Figure 1 was verified by photocatalytic voltammetry of a coated electrode in stirred electrolyte. Under illumination, a photocathodic wave (*J*_light_) with an onset of 0.3 V was observed, followed by a second onset beginning at 0.1 V vs. SHE (Figure 2b). A voltammogram in the dark (*J*_dark_) revealed a reduction wave which was subtracted from *J*_light_ to give the photocatalytic wave representing light‐driven electron transfer (*J*_cat_). The derivative of *J*_cat_ (Figure 2c) revealed two half‐wave potentials (*E*_hw_) of +0.22 V and 0.0 V vs. SHE, in close agreement with the midpoint potentials (*E*_m_) of cyt *c* and Q_0_, respectively. Further verification of the mechanism came from an action spectrum of external quantum efficiency (EQE—charge carriers/incident photons). Peaks in this spectrum showed good alignment to bands in the absorbance spectrum of the RC‐LH1 complex (Figure 3a), identifying it as the source of the photocurrent. Comparison between the EQE action spectrum and a (1−T) % absorptivity spectrum revealed significant disparities, however, stemming from the nature of light management within the device (see Figure S6 and accompanying text for more detail).

The *J*_peak_ and EQE were also determined across a range of actinic light intensities from 0.0008 to 80 mW cm^−2^ (Figure 3b). The maximum EQE of 57±6 % at low light irradiance (3.6 μW cm^−2^/874 nm) is one of the highest reported EQEs to date. This value decreased at illumination intensities above 0.1 mW cm^−2^, an effect that likely stemmed from electron transfer bottlenecks and short‐circuiting processes which were exacerbated at high RC excitation rates (see Figure S6b and accompanying text for more detail). These loss processes form a target for rational design strategies to improve biophotoelectrode performance.

**Figure 4:**
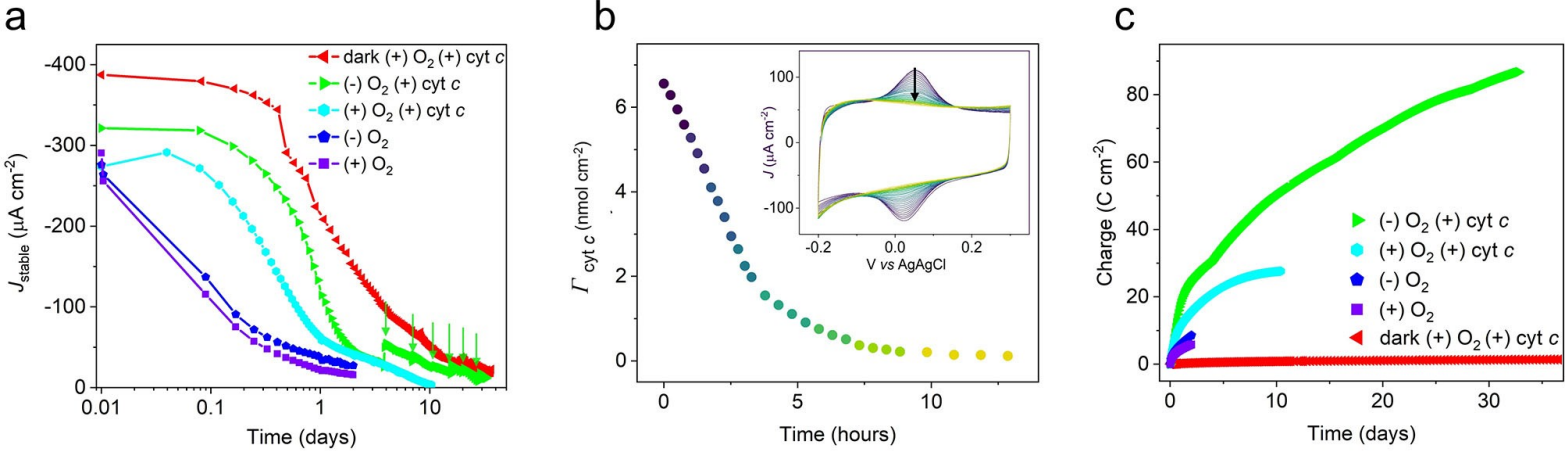
Operational stability. a) Variation of *J*_stable_ over 33 days in the presence (+) and absence (−) of oxygen and cyt *c*. For dark conditions, a photocurrent was measured with the excitation light on for 2 minutes every 2 hours (98.3 % dark) for the first 24 hours, followed by 2 minutes for every 4 hours thereafter (99.2 % dark). Otherwise, the excitation light was on for 95 % of the time. Arrows indicate replacement of the electrolyte. A linear scale plot is reported in Figure S11b. b) Desorption of cyt *c* as determined by changes in the area under cyclic voltammogram peaks (inset). Scan rate was 250 mV s^−1^. c) Cumulative charge transfer.

Operational stability of the IO‐mITO|cyt *c*|RC‐LH1 electrodes was examined under near‐continuous high‐light (42.3 mW cm^−2^) illumination (cycles of 57 minutes on, 3 minutes off), in the presence or absence of oxygen. Over 48 hours the value of *J*_stable_ decayed by around 90 % both in the presence (+) and absence (−) of oxygen (Figure 4a, purple and blue, respectively). The rate of decay was marginally faster in the presence of oxygen than in its absence, with a half‐life of just below and above 2 hours, respectively. It was concluded that the reversible desorption of the electron transfer relay, cyt *c*, from the electrode was primarily responsible for the loss in *J*_stable_ under these conditions as measurements showed that the amount of cyt *c* loaded on the electrode decreased with a similar half‐life of ≈2 hours (Figure 4b). This was confirmed by regenerating the cyt *c* film at the end of such a two‐day period via immersion of the electrode in a 200 μM cyt *c* solution for 5 minutes that restored 81 % of the initial *J*_stable_ (Figure S7a). However, for electrodes operated in air, regenerating the cyt *c* film resulted in only a 65 % recovery of *J*_stable_ (Figure S7b), indicating an additional irreversible decay component that we attribute to damage from reactive oxygen species (ROS).

To stabilize the interfacial electron transfer pathway during operation, 20 μM cyt *c* was added to the working electrolyte. This extended the operational half‐life from ≈2 hours to ≈9 hours in presence of oxygen (Figure 4a, cyan). The addition of cyt *c* to the working electrolyte also amplified the beneficial effect of excluding oxygen, extending operational half‐life to ≈20 hours, and producing photocurrents that decayed to 5 μA cm^−2^ after 10 days. The extracted half‐lives of operational stability under various electrolyte conditions are summarized in Table S2.

An absorbance spectrum of a cyt *c*‐containing electrolyte taken either before (Figure S8a) or after (Figure S8b) 48 hours of near‐continuous illumination in the absence of oxygen revealed a shift in the ratio of oxidized to reduced Q_0_ in favor of the latter, suggesting depletion of the RC electron acceptor in the bulk electrolyte could also be partially responsible for the decay in *J*_stable_. This prompted an experiment in which the electrolyte was replenished every few days thereafter, producing a partial restoration of the photocurrent and enabling it to be maintained at a level of 10–20 μA cm^−2^ for 33 days under near‐continuous high‐light illumination (Figure 4a, green). This represents a record in the continuous operation of a biohybrid electrode based on a photosynthetic enzyme, surpassing by roughly 50‐fold the previous benchmark of a PSI biohybrid that operated for 16 hours when illuminated ≈30 % of the time,^[21]^ and by more than 12‐fold a previous bRC benchmark of 65 hours.^[22]^ Furthermore, the total charge transferred over the recorded operation of the electrode was 86 C cm^−2^ (Figure 4c), 45‐fold greater than a previous benchmark with RC‐LH1 complexes.^[11]^ These intense light conditions (46 mW cm^−2^/870 nm LED) resulted in approximately 2.2 billion excitations per RC‐LH1 complex per month, simulating the number of excitations the complex would experience under a full year of solar irradiance in the Netherlands.^[31]^ Considering that the photocurrents plateaued at approximately 15 mW cm^−2^ (Figure 3b), which is much greater than the solar insolation of the Netherlands, we postulate that stability may be improved under less intense light‐conditions, as the bRC could more efficiently process incoming excitations into desired photochemistry before they are lost in deleterious side‐reactions.

To investigate loss mechanisms not linked to photochemistry, electrodes were operated under aerobic conditions in the presence of cyt *c*, but in the dark apart from brief exposure to light every four hours to probe photocurrent activity. This revealed a decay in *J*_stable_ that was similar to that seen under continuous illumination under anaerobic conditions over the same time period (Figure 4b, red compared with green). This indicated a major decay mechanism not connected to photocatalysis or ROS which we attribute to fouling of the electrode. Electrode fouling is a commonly encountered problem in electrochemical biosensors, resulting from the irreversible and nonspecific adsorption of proteins to,[^32^, ^33^] and insulation of, the electrode surface.^[34]^ In our case, the irreversible electrode adsorption of cyt *c* would inhibit fast interfacial electron transfer with the electrode[^32^, ^35^] and restrict any rotational mobility needed for electron relay to the RC.^[25]^ Evidence for electrode fouling by cyt *c* was observed in the slowed rates of photocurrent onset (Figure S9) and interfacial electron transfer (Figure S10, see supporting text for more detail) after 10 days of operation in a working electrolyte containing cyt *c*. Further support stemmed from the recoverable photocurrents (the photocurrent after the cyt film was regenerated) whereby photocurrents after 10 days of operation were approximately 60 % larger when cyt *c* was absent from the working electrolyte (Figure S11). Lastly, refreshing the electrolyte did not result in any substantial improvement in photocurrent for electrodes operated in the dark (Figure S11b, red arrows), in contrast to findings for electrodes under near‐continuous illumination (Figure 4b and S11b, green arrows). Turning to the photoprotein, a notable loss in functional LH1 (95 %) was observed in the EQE action spectrum after 30 days of operation in the light (Figure S12a), which we partially attribute to the decoupling of the LH1 from the RC, since the spectral signature of the LH1 ring was not proportionally decreased in the 1‐Reflectance spectrum of the electrode (Figure S12b). The loss in functional LH1 was also observed for the electrode operated for 30 days in the dark, which we attribute to self‐shading from unwired RC‐LH1 complexes (see Figure S12b and accompanying supporting text for more detail). Degradation of LH1 cofactors may have also contributed to the loss in LH1 phenotype, with pigment extractions revealing formation of a photo‐degradation product which we attribute to 3‐acetyl‐chlorophyll *a* ^[36]^ (see Figure S13 and accompanying text).

**Figure 5:**
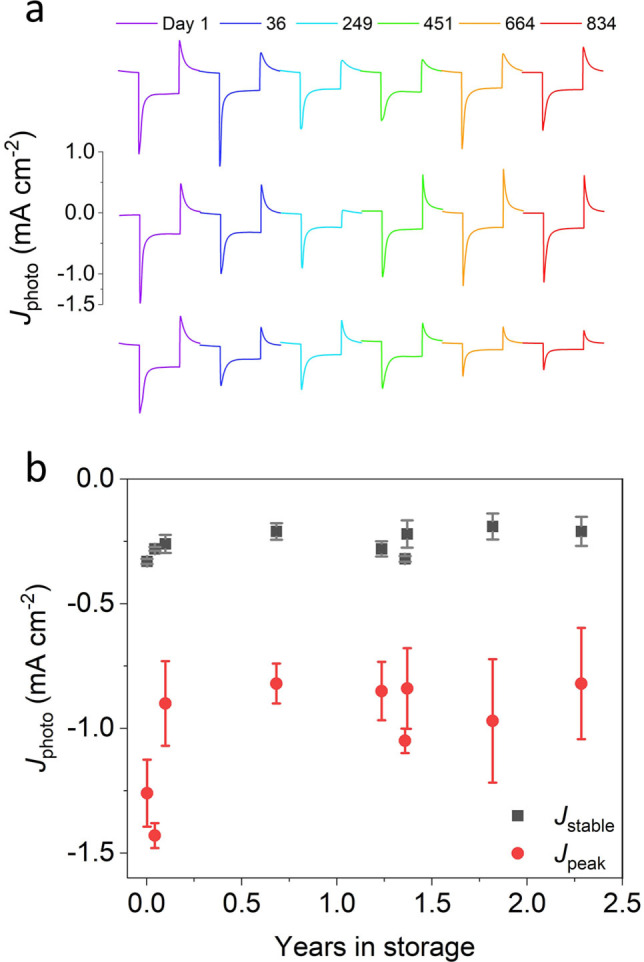
Electrode stability in storage. a) Two minutes light on/off transients at six intervals over 834 days. Electrodes were stored at 4 °C in the dark in 20 mM Tris (pH 8.0) between measurements. b) Variation of peak and stable photocurrent during long‐term storage for 2.3 years.

Photocurrent output was also assessed at intervals for electrodes stored in the dark at 4 °C in a buffer lacking cyt *c* (20 mM Tris pH 8.0/50 mM KCl). The ionic strength of the buffer was sufficient to desorb the cyt *c* film during storage, which was regenerated before measurement. Example light‐on/off photocurrent traces recorded for three electrodes at intervals over 834 days are shown in Figure 5a. Although the peak photocurrents obtained from electrodes declined by about a third over the first couple of months of storage, thereafter they remained very stable, and *J*_stable_ remained remarkably consistent for over two years of storage (Figure 5b).

To summarize these data, relatively high photocurrents could be generated using freshly prepared biophotoelectrodes comprising an IO‐mITO matrix suffused with purple bacterial RC‐LH1 proteins and cyt *c*, with peak photocurrents of over 4 mA cm^−2^ after optimization, and steady‐state photocurrents of routinely around 0.25–0.3 mA cm^−2^. Several factors were seen to cause a decline in steady‐state photocurrent over time for electrodes under continuous assessment. Over the period of many hours, desorption of cyt *c* from the IO‐mITO matrix appeared to cause a relatively rapid drop in *J*_stable_ that could be mitigated by including cyt *c* in the electrolyte, which stabilized the electrical connection between the photoactive RC‐LH1 complexes and the IO‐mITO electrode. The exclusion of oxygen was also beneficial, particularly when cyt *c* was also present, as was replenishment of the electrolyte to restore a maximal oxidized/reduced ratio of quinone for charge collection from the RC‐LH1 complexes.

Over the period of a month, however, there was steady decline in *J*_stable_ to around 5 % of its initial value. Albeit at somewhat different initial rates, this decline was seen both for electrodes under near‐continuous (95 % of the time) illumination and for electrodes under near‐continuous (99.17 % of the time) dark conditions (Figure 4a). This suggested that a light‐independent process became limiting during this time. A candidate for this process is the gradual inactivation of the cyt *c* film. Although it was shown that addition of cyt *c* to the buffer partially addressed the rapid initial drop in *J*_stable_ over the first day, it is striking that the highly characteristic cyt *c* peak seen at 550 nm in 1‐Reflectance spectra of fresh electrodes (Figure 1c), and the corresponding trough seen in EQE spectra of photocurrents from fresh electrodes (Figure 3a) was not seen in equivalent spectra for electrodes operated for 10 or 30 days (Figure S12). This could indicate gradual irreversible loss of the wiring cyt *c* layer from the electrode matrix, despite the presence of cyt *c* in solution. Taken together with the slowed photocurrent onset and interfacial electron transfer rates and decreased cyt *c* loading after prolonged operation in the presence of aqueous cyt *c* (Figure S10), these observations points towards loss of electron transfer pathways stemming from electrode fouling as the main limitation for continuous operation.

Perhaps the most encouraging finding was that the *J*_stable_ produced by these electrodes was essentially invariant over two years of storage (Figure 5b), at a level of around 0.25 mA cm^−2^. Our reported stability in storage surpasses previous records of PSI biohybrid stability without any loss of activity under intermittent testing for over 85 days,^[37]^ as well as records of biophotovoltaic stability in storage measured over 281 days, with photocurrents around 3 μA cm^−2^.^[38]^ Previous studies that wired bRCs to bare metal electrodes using cyt *c* likely suffered from accelerated failure of the interfacial ET pathway due to fouling of the electrode by cyt *c*, which over time has been shown to strongly adsorb to bare metal electrodes in non‐functional orientations^[35]^ or even denature.[^32^, ^33^] Utilizing a hydroxyl functionalized mITO electrode, as well as desorbing cyt *c* from the electrode surface, likely played a crucial role in preventing electrode fouling during storage. These stabilities indicated that, although not yet suited to applications requiring prolonged light exposure, such biohybrid electrodes could be suited to applications involving short term exposure over a time scale of minutes or a few hours. A potential candidate could be sensing, and the potential of light‐powered RC and RC‐LH1 electrodes for applications such as herbicide biosensing,[^39^, ^40^, ^41^] and touch sensing[^42^, ^43^] are being been explored. Although such applications would not necessarily require electrodes exhibiting long‐term photocurrent stability, involving relatively brief measurements, they would require electrodes capable of being stored for long periods before use,[^19^, ^44^] and the data summarized in Figure 5b were promising in this regard.

Returning to operation under more demanding conditions, several improvements to electrode design can be contemplated. The IO‐mITO matrix provided a means of supporting functional photoproteins for years, but although initially very effective, the use of cyt *c* to “wire” the RC‐LH1 complexes to the electrode was problematic. Clearly a more robust wiring moiety that matches photoprotein stability would be beneficial, particularly if it also resisted fouling the electrode. Furthermore, it would be beneficial to address the stability of the photoproteins themselves. As illustrated in Figure 4a, one option would be to exclude oxygen as its presence does accelerate drops in current over time, in agreement with previous findings.^[21]^ Another option might be to utilize naked RCs rather than the larger RC‐LH1 complexes. Although the latter have a higher light harvesting capacity, and tend to produce higher photocurrents when compared side‐by‐side, the LH1 system does seem more prone to photodamage and so an option may be to utilize higher densities of the “smaller but tougher” RC photodiode for applications requiring prolonged exposure of electrodes to high light intensities, rather than the larger but more fragile RC‐LH1 complex that has evolved for solar energy conversion in relatively low light environments where efficient light harvesting is at a premium. Of course, stabilizing electron transfer pathways that channel RC‐LH1 excitations into desired photochemistry rather than competing photodamaging side‐reactions may also resolve the RC‐LH1 degradation observed in this work.

## Conclusion

Our study explored the durability of photoprotein function in an abiotic, biohybrid environment. We found that the photoprotein outlasted the electron transfer pathways which wired it to the electrode, and that by preserving these pathways, we were able to extend continuous biophotoelectrode activity to one month under intense continuous light, which simulated a full year of solar insolation in the Netherlands. By preventing electrode fouling during storage, we were able to preserve over 70 % of electrode activity for over two years. Such a high stability platform is suitable for biosensor applications that demand long storage times but shorter operation times. However, operational stability in biophotovoltaic operation, which demand function under continuous light for years, will require further investigation to overcome electrode fouling encountered at these hitherto unencountered time domains. Nevertheless, we have demonstrated that, under the right conditions, photoproteins can operate for years in biohybrid devices. These insights will help shape the rational design of biohybrid devices with performances and stabilities necessary for biotechnological applications.

## Conflict of interest

The authors declare no conflict of interest.

1

## Supporting information

As a service to our authors and readers, this journal provides supporting information supplied by the authors. Such materials are peer reviewed and may be re‐organized for online delivery, but are not copy‐edited or typeset. Technical support issues arising from supporting information (other than missing files) should be addressed to the authors.

Supporting Information

## Data Availability

The data that support the findings of this study are available from the corresponding author upon reasonable request.
